# Multivalent, calcium-independent binding of surfactant protein A and D to sulfated glycosaminoglycans of the alveolar epithelial glycocalyx

**DOI:** 10.1152/ajplung.00283.2023

**Published:** 2024-02-20

**Authors:** Rabia Avcibas, Anna Vermul, Vladimir Gluhovic, Nico Boback, Raquel Arroyo, Paul Kingma, Miriam Isasi-Campillo, Lucia Garcia-Ortega, Matthias Griese, Wolfgang M. Kuebler, Matthias Ochs, Daniel Lauster, Elena Lopez-Rodriguez

**Affiliations:** ^1^Institute of Functional Anatomy, Charité – Universitätsmedizin Berlin, Berlin, Germany; ^2^Division of Neonatology and Pulmonary Biology, Cincinnati Children’s Hospital Medical Center, Cincinnati, Ohio, United States; ^3^Department of Biochemistry and Molecular Biology, Complutense University Madrid, Madrid, Spain; ^4^Dr. von Hauner Children’s Hospital, University Hospital, LMU Munich, German Center for Lung Research, Munich, Germany; ^5^Institute of Physiology, Charité – Universitätsmedizin, Berlin, Germany; ^6^German Center for Cardiovascular Research (DZHK), Berlin, Germany; ^7^Keenan Research Centre, St. Michael’s Hospital, University of Toronto, Toronto, Ontario, Canada; ^8^Department of Surgery, University of Toronto, Toronto, Ontario, Canada; ^9^Department of Physiology, University of Toronto, Toronto, Ontario, Canada; ^10^German Center for Lung Research (DZL), Berlin, Germany; ^11^Core Facility Electron Microscopy, Charité – Universitätsmedizin Berlin, Berlin, Germany; ^12^Institute of Pharmacy, Biopharmaceuticals, Freie Universität Berlin, Berlin, Germany

**Keywords:** alveolar epithelial glycocalyx, binding affinity, glycosaminoglycans, lung collectins, multivalent binding

## Abstract

Lung surfactant collectins, surfactant protein A (SP-A) and D (SP-D), are oligomeric C-type lectins involved in lung immunity. Through their carbohydrate recognition domain, they recognize carbohydrates at pathogen surfaces and initiate lung innate immune response. Here, we propose that they may also be able to bind to other carbohydrates present in typical cell surfaces, such as the alveolar epithelial glycocalyx. To test this hypothesis, we analyzed and quantified the binding affinity of SP-A and SP-D to different sugars and glycosaminoglycans (GAGs) by microscale thermophoresis (MST). In addition, by changing the calcium concentration, we aimed to characterize any consequences on the binding behavior. Our results show that both oligomeric proteins bind with high affinity (in nanomolar range) to GAGs, such as hyaluronan (HA), heparan sulfate (HS) and chondroitin sulfate (CS). Binding to HS and CS was calcium-independent, as it was not affected by changing calcium concentration in the buffer. Quantification of GAGs in bronchoalveolar lavage (BAL) fluid from animals deficient in either SP-A or SP-D showed changes in GAG composition, and electron micrographs showed differences in alveolar glycocalyx ultrastructure in vivo. Taken together, SP-A and SP-D bind to model sulfated glycosaminoglycans of the alveolar epithelial glycocalyx in a multivalent and calcium-independent way. These findings provide a potential mechanism for SP-A and SP-D as an integral part of the alveolar epithelial glycocalyx binding and interconnecting free GAGs, proteoglycans, and other glycans in glycoproteins, which may influence glycocalyx composition and structure.

**NEW & NOTEWORTHY** SP-A and SP-D function has been related to innate immunity of the lung based on their binding to sugar residues at pathogen surfaces. However, their function in the healthy alveolus was considered as limited to interaction with surfactant lipids. Here, we demonstrated that these proteins bind to glycosaminoglycans present at typical cell surfaces like the alveolar epithelial glycocalyx. We propose a model where these proteins play an important role in interconnecting alveolar epithelial glycocalyx components.

## Graphical abstract

**Figure:**
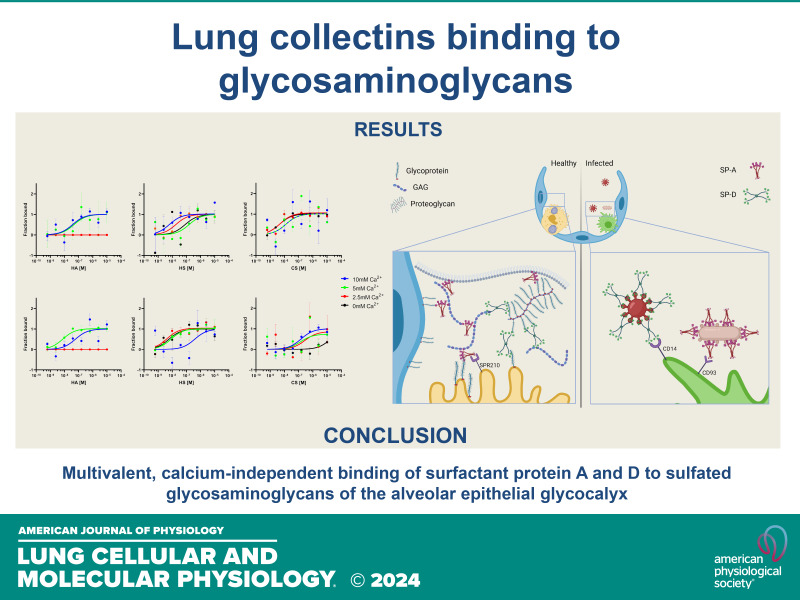

## INTRODUCTION

The alveolar epithelium is composed of two types of epithelial cells, alveolar epithelial type I and II cells, which as epithelial cells produce a “sugar-coat” or glycocalyx at their apical side (1–5). A cell glycocalyx is defined by three components: glycoproteins and proteoglycans, which may be anchored to the cell membrane or proteins, and free glycans or glycosaminoglycans (GAGs). In addition, on top of the alveolar epithelium, there is a liquid lining layer, consisting of a hypophase and a surface surfactant film, where lung surfactant exerts its surface tension-lowering activity (6, 7). The alveolar epithelial glycocalyx has, therefore, a special location that predisposes it for a potential interaction with lung surfactant components (2, 8, 9). Both components, the alveolar epithelial glycocalyx and lung surfactant, are therefore involved in the barrier function of the alveolar epithelium.

GAGs are typically classified in accordance to the difference of repeating disaccharides into four groups: heparin (HP)/heparan sulfate (HS)-containing n-acetyl-glucosamine (GlcNAc) and glucuronic acid (GlcA), chondroitin sulfate (CS)/dermatan sulfate (DS)-containing n-acetyl-galactosamine (GalNAc) and GlcA, keratan sulfate (KS)-containing GlcNAc and galactose (Gal), and the nonsulfated hyaluronan (HA)-containing GlcNAc and GlcAc (10). GAGs have been found to vary with lung diseases, such as acute respiratory distress syndrome (ARDS), cystic fibrosis, asthma, emphysema, sarcoidosis, idiopathic pulmonary fibrosis (IPF), and animal models of lung disease, such as bleomycin-induced lung injury (11–14) and ventilation-induced lung injury (12, 15). Shedding of GAGs from the glycocalyx is used as a biomarker of epithelial barrier injury (9, 16) during lung disease.

Lung surfactant, on the other hand, is a mixture of lipids and proteins with the main function of decreasing surface tension. Two surfactant proteins, surfactant protein A (SP-A) and D (SP-D) are predominantly, but not exclusively found in the lung (17–21), where SP-A is one of the most abundant surfactant proteins, accounting for around 5–6%, and SP-D only in less than 0.5% (22–24). For example, SP-A and SP-D have been found at the RNA or protein level in human esophagus, stomach, small intestine, large intestine, liver, gall bladder, pancreas, thymus, and placenta (25–30). These two proteins are classified as C-type lectins (CTLs) and more specifically as collectins, as they also contain a collagen domain in their structure. As C-type lectins, they are by definition Ca^2+^-dependent glycan-binding proteins with a typical carbohydrate recognition domain (CRD). CTLs such as collectins, selectins, and others, oligomerize forming big protein complexes that offer multiple CRD, and therefore, binding sites for multivalent ligands (which offer multiple ligand units within the same molecule). This oligomerization phenomenon hence increases the avidity of the protein for these multivalent ligands (31).

SP-A and SP-D interact through their collagen domain to form trimeric units, which oligomerize through their N-terminal domain. SP-A typically oligomerizes to octadecameric structures, similar to a bouquet of flowers, whereas SP-D oligomerizes into dodecameric cruciform-like structures, which can further form “stellate multimers” (32). This multimerization increases the overall avidity of binding to carbohydrates, enhancing their ability for pathogen opsonization and agglutination. In addition, high oligomerization may increase their binding avidity to multivalent ligands, such as GAGs, which offer repetitive units (monosaccharides) to bind to. However, whether SP-A and SP-D can bind to GAGs is yet a question to be answered.

Here, we hypothesized that lung collectins, SP-A and SP-D, may interact with GAGs typically found at the alveolar epithelial glycocalyx. To test this hypothesis, we first validated the binding of these collectins to monosaccharides by the use of microscale thermophoresis (MST) and then used the same method to measure binding to GAGs. In this way, SP-A and SP-D may not only bind pathogens through their wall sugars but also GAGs found in the alveolar epithelial glycocalyx. As such, they may form an integral part of the alveolar epithelial glycocalyx under healthy conditions and absence of pathogens, keeping all elements, carbohydrate and lipid-based components, together.

In this study, we have used microscale thermophoresis (MST) to measure the binding of SP-A and SP-D to sugars and glycosaminoglycans and to calculate dissociation constants (K_D_) (33–36). In addition, we validated the binding with indirect methods and systematically investigated the calcium dependency. To understand the physiological importance of this binding, we quantified the amount of free (or shed) GAGs in bronchoalveolar lavage (BAL) and analyzed the ultrastructure of the alveolar glycocalyx of mice lacking one of the two proteins in vivo.

## MATERIALS AND METHODS

Human bronchoalveolar lavage (BAL) fluid of patients with pulmonary alveolar proteinosis (PAP) was obtained from *1*) the Hospital “Fundación Jimenez Diaz,” Madrid, Spain, and protocols were approved by the ethics committee of the hospital and informed consent was signed by the donor; and *2*) from a patient with PAP due to disease-causing mutations in CSF2RA as previously described (37) at Klinikum der Universität München, with the ethical approval of the University (EK111-13, EK20-329).

Human recombinant SP-A1 (OPCD07130) and SP-A2 (OPCD07132) were purchased from Aviva Systems Biology (San Diego). Human recombinant SP-D was produced and isolated as described before (38, 39) in the Division of Neonatology and Pulmonary Biology, Cincinnati Children’s Hospital Medical Center. Details about the proteins used in this study can be found in Table 1.

**Table 1.** Details about the source of the proteins used in this study

Protein	Concentration	Source	Reference Number	Provider
recSP-A1	20 µM	Recombinant, prokaryotic expressed	OPCD07130	Aviva Bio Systems
recSP-A2	20 µM	Recombinant, prokaryotic expressed	OPCD07132	Aviva Bio Systems
rhSP-D	7.44 µM	Recombinant, CHO cells (eukaryotic, mammal)	AT-100	Airway Therapeutics
hSP-A	6.5 µM	Human PAP patient, BAL isolated		
hSP-D	0.6 µM	Human PAP patient, BAL isolated		

The following sugars and GAGs were used in this work: chondroitin sulfate sodium salt (456280050, Acros Organics, Beel, Belgium); d(−)fructose (F/1950/50, Fisher Reagents, Schwerte, Germany); l-fucose (225880010, Acros Organics, Beel, Belgium; d(+)-galactose (150611000, Acros Organics, Beel, Belgium); N-acetyl-d-galactosamine (J66095, AlfaAesar, Kandel Germany); l(−)-glucose (241920010, Acros Organics, Beel, Belgium); d-glucuronic acid (L14350, AlfaAesar, Kandel Germany); N-acetyl-d-glucosamine (A13047, AlfaAesar, Kandel, Germany); hyaluronic acid sodium salt (J6693, AlfaAesar, Kandel, Germany); heparan sulfate sodium (H7640, Sigma Aldrich, Darmstadt, Germany); d(+)-maltose (329911000, Acros Organics, Beel, Belgium); d(+)-mannose (150600258, Acros Organics, Beel, Belgium); N-acetyl-d-mannosamine monohydrate (L11167, AlfaAesar, Kendal, Germany); and N-acetyl-neuraminic acid (A2388, Sigma Aldrich, Darmstadt, Germany). Details about the GAGs used in this study can be found in Table 2.

**Table 2.** Details about the source of the GAGs used in this study

GAG	Size	Source	Reference Number	Provider
HA	1-2 Mio Da	Streptococcus equi	J6693	AlfaAesar
HS	Not known	Bovine kidney	H7640	SigmaAldrich
CS	Not known	Porcine intestinal mucosa	456280050	Acros Organics

CS, chondroitin sulfate; GAG, glycosaminoglycan; HA, hyaluronan; HS, heparan sulfate.

Healthy wild-type (WT) (C57BL/6, *n* = 5), SP-A^−/−^ (SP-A KO, *n* = 9) and SP-D^−/−^ (SP-D KO, *n* = 10) mice were used in this study. Bronchoalveolar lavage (BAL) fluid was obtained after euthanasia of the animal following standard procedures approved by local governmental authorities (State Office for Health and Social Affairs Berlin, LAGeSo, Germany). BAL fluid was obtained by cannulation of the trachea, followed by a thoracotomy. The pulmonary vasculature was then rinsed via the right ventricle with 0.9% NaCl. Subsequently, two 1.5 mL aliquots of 0.9%NaCl were instilled through the cannula into the lungs. The liquid was withdrawn and collected in 15 mL tubes. Cell-free BAL fluid was obtained after cells were pelleted and discarded by centrifugation at 300 *g* for 5 min at4°C.

### Isolation of hSP-A

SP-A was isolated from BAL fluid from PAP patients using a sequential butanol and octylglucoside extraction modified from the original protocol of Hawgood and colleagues 1987 (40). Briefly, 500 mL of BAL fluid from a PAP patient was centrifuged at 100.000 *g* for 1 h at 4°C, and the pellet containing surfactant complexes was stored at -20°C until further use. The pellet was dissolved in 20 mL 0.9% NaCl and protein concentration was determined using the MicroBCA Protein Assay Kit (23235, Thermo Scientific). The protein solution was then added, drop by drop, to 30 mL of butanol per milligram of protein, under constant stirring, followed by a 30 min incubation under constant stirring. After centrifugation of the solution at 5,000 *g* for 30 min at 15°C, precipitated non-soluble components in the pellet were eliminated and the supernatant was dried under compressed air. The dried residue was homogenized in OGP-buffer, containing 20 mM octyl-D-glucopyranoside (230340050, Acros), 10 mM HEPES, 150 mM NaCl and centrifuged at 42.100 rpm (Rotor type 70Ti), for 30 min, at 15°C. The supernatant was discarded and the pellet homogenized in 5 mM Tris buffer (pH 7.4). Later, the protein was dialysed in the same buffer using a membrane of a 3500 MWCO (Slide-A-Lyzer Dyalisis Cassette 66110, Thermo Scientific) to remove detergent traces. After a last centrifugation step [42.100 rpm (Rotor type 70Ti), for 30 min, at 15°C] to precipitate and eliminate aggregates, the protein concentration was determined with a BCA Protein Assay kit. For MST experiments, molar concentration of the protein was calculated based on the molecular weight of the (36 kDa).

### Isolation of hSP-D

One liter of cell-free BAL fluid was ultracentrifuged at 100.000 *g* for 1 h at 4°C to clarify supernatant from membranous complexes and pulmonary surfactant. The supernatant solution was adjusted to 20 mM Tris-HCl, 5 mM EDTA pH 7.4, and filtered through 0.45 µm pore membranes. CaCl_2_ was added to yield a final concentration of 10 µM and supernatant was incubated for 16 h at 4°C with 5 mL of Superdex 75 prep grade resin (S6657, Sigma-Aldrich, St. Louis) equilibrated in 20 mM Tris-HCl, 150 mM NaCl, 10 mM CaCl_2_ pH 7.4. SP-D was eluted after extensive washing with 20 mM Tris-HCl, 1 M NaCl, 10 mM CaCl_2_ pH 7.4, employing 20 mM Tris-HCl, 50 mM MnCl_2_ pH 7.4. Fractions containing SP-D were analyzed by SDS-PAGE, pooled together, and dialyzed against 20 mM Tris-HCl, 200 mM NaCl, 1 mM EDTA pH 7.5, then stored at −80°C. Protein purity was checked by SDS-PAGE and Western blot using mouse anti-SP-D monoclonal antibody (2D12A88, Seven Hills Bioreagents; Cincinnati, OH). SP-D concentration was calculated by absorption spectroscopy, considering the following extinction coefficient at 280 nm: E^0.1%^ = 0.81 (mg^−1^ × mL × cm^−1^). For MST experiments, molar concentration of the protein was calculated based on the molecular weight of the (43 kDa).

### SDS-PAGE and Coomassie Blue Staining

Protein samples were prepared by mixing with Laemmli buffer 4X at a final concentration of 100 µg/mL and heated for 10 min at 100°C. Polyacrylamide gels (10%) were loaded with a molecular weight standard and 10 µL of protein sample per well. After gel electrophoresis, the gel was stained after fixing (with 25% isopropanol and 10% acetic acid solution) for 15 min following provider instructions with PAGEblue Protein Staining Solution (24620, Thermo Fisher Scientific, Massachusetts). Briefly, after washing the gel three times for 10 min with distilled water, the gel was incubated under agitation with 20 mL of PAGEblue solution for 1 h at room temperature. Destaining of the gel was achieved by washing the gel with distilled water two times for 5 min at room temperature.

### Immunodetection of SP-A and SP-D

After gel electrophoresis, as explained in *SDS-PAGE and Coomassie Blue Staining*, the proteins were transferred to a PVDF membrane (1620177, Bio-Rad Laboratories, Feldkirchen, Germany). The membranes were blocked with 5% milk (T145.1, Carl Roth, Karlsruhe, Germany) in Tris Buffer + 0.1% Tween-20 (TBS-T) for 1 h at room temperature, and incubated overnight with either anti-SP-A antibody (ab190087, Abcam, Cambridge, UK) at a final concentration of 1 µg/mL or anti-SP-D (AF1920, R&D Systems, Abingdon, UK) at a final concentration of 1 µg/mL in 5% milk in TBS-T. After washing the membranes with TBS-T, the secondary antibody, donkey anti-goat-HRP (ab6885, Abcam, Cambridge, UK) at a final concentration of 0.1 µg/mL in 5% milk in TBS-T, was applied and incubated for 1 h at room temperature. The specific immune-detected signal was developed using an ECL Western Blot detection reagent (GERPN2106, Cytiva, Freiburg, Germany) and visualized in a Chemostar imaging system (Intas Science Imaging Instruments, Göttingen, Germany).

### Labeling of SP-A and SP-D

Human-isolated and recombinant proteins were labeled using the Monolith Protein Labeling Kit RED-NHS according to the manufacturer’s instructions. This kit reagent reacts with lysine residues present in the sequence of the protein, as the dye carries a reactive NHS-ester group. Briefly, as all the proteins were in a Tris buffer solution, the buffer solution was exchanged to a HEPES-based buffer with the A-Column provided in the kit. Then, 10 µM solution of protein was incubated with a threefold excess of dye in 100 µL for 30 min at room temperature protected from light. The labeled protein was separated from the free dye using the B-Column provided in the kit. The concentration of the labeled protein {c(M) = [A_205_ − (A_650_ × 0.19)]/[31 × MW_protein_]) and the degree of labeling [DOL = A_650_/(195.000 M^−1^cm^−1^ × c(M)] were calculated according to the manufacturer’s instruction, measuring absorption at 205 nm (for the protein) and 650 nm (for the dye).

### Sample Preparation and Settings for Microscale Thermophoresis

Thermophoresis is the phenomenon of movement of particles along a temperature gradient. Solvated molecules move from higher to lower temperature. This movement or depletion of molecules from the heat is quantified by the Soret coefficient S_T_: C_hot_/C_cold_ = exp (−S_T_ΔT). The depletion of molecules depends on the interface between molecule and solvent. Therefore, thermophoresis monitors changes in size, charge, and solvation entropy of molecules. In this regard, the thermophoresis or movement of a protein is different from a protein-ligand complex (35). Microscale or miniaturized thermophoresis allows to monitor molecular interaction in only a few microliters. For this method, the fluorescently labeled protein is mixed with a decreasing concentration of a ligand in 16 glass capillaries. The initial fluorescence is measured (F_cold_), then an infrared (IR) laser beam is switched on and focused on the center of the first capillary, where the temperature gradient is created from the center to the walls of the capillary. At the same time, a dichroic mirror allows fluorescence from the labeled protein to be monitored (F_hot_). The rate of fluorescence change is related to the amount of protein-ligand complex form, which depends on the ligand concentration, due to changes in size, charge, or hydration shell of the protein-ligand complex. The dissociation constant K_D_ is calculated from the binding curve derived by plotting the normalized fluorescence (F_Norm_) at a given time after the start of the thermophoresis against the respective ligand concentration (33, 34, 36).

Samples were measured in a Monolith NT.115, using Premium Capillaries (MO-K025) and Monolith Protein labeling kit RED-NHS (MO-L011) all from NanoTemper Technologies (Munich, Germany). Solutions of a 3X concentrated MST buffer (15 mM Tris, 300 mM NaCl, 0.3% Tween 20, 15 mM CaCl_2_, pH 7.4), sugars (270 mM) or GAG (30 µM) solution, and protein solution (27 nM) were prepared separately. When a different concentration was used, a threefold concentration of the solution was prepared. Afterward, sugars and GAG solutions were diluted 1:2 with water in a series of 16 steps. For the measurement, 3 µL of each prepared component (3 µL MST buffer, 3 µL GAG solution, and 3 µL labeled protein) was mixed, centrifuged shortly to eliminate precipitates, and loaded into a glass capillary. The final concentrations of the sugars were thus between 90 –and 0.00275 mM or GAGs 10 and 0.000305 µM for GAGs, respectively. The 16 capillaries were loaded in the Monolith NT.115 and measurements were performed with a 40% LED power and medium MST power at 25°C.

### Interferometric Light Scattering Microscopy

Interferometric light scattering microscopy (iSCAM) measurements were performed with the OneMP (Refeyn), equipped with a 525 nm laser. The device was isolated from environmental vibrations by using the Accurion (i4 Series) active vibration isolator. Protein samples were diluted with sodium and magnesium-free DPBS buffer (pH 7.4) to concentrations (15–400 nM) that yielded the optimal signal separation and number of events. The samples (5 µL) were loaded onto the glass coverslip by mixing into a buffer droplet (15 µL). All measurements were performed at 19–20°C in a regular field of view using the auto-focus function. The resulting contrasts were converted into masses using the NativeMarkMT (ThermoFisher). Spectra were plotted in DiscoverMP.

### Binding Inhibition Assay

Following previously described assays (41, 42), 96-well plates (83.3924.005, Sarstaedt AG, Nümbrecht, Germany) were coated with 70 ng/well (total volume of 100 µL/well) of mannan (M7504, Sigma-Aldrich, St. Louis) dissolved in 0.1 M NaHCO_3_ buffer (pH 9.6) overnight at 4°C. After removal of the coating solution, wells were blocked with 100 µL of 5 mM Tris with 2% BSA (Serva, Heidelberg, Germany) at room temperature for 2 h. Plates were washed three times with 100 µL/well washing buffer (5 mM Tris, 0.9% NaCl, 0.05% Tween-20, and 5 mM CaCl_2_). Next, different concentrations of HA, HS, or CS ranging from 15 µM to 30 nM and hSP-A at 14 ng/well were added to washing buffer with 0.1% BSA. The plate was incubated for 2 h at room temperature to allow the protein to bind to either mannan or GAGs. After subsequent washing, as stated before, mannan-bound hSP-A was immunodetected by incubation with a specific anti-SP-A antibody (ab190087, Abcam, Cambridge, UK) at a 0.5 µg/mL concentration for 2 h at room temperature in washing buffer. After washing the excess of antibody, a secondary antibody conjugated with HRP (horseradish peroxidase) anti-goat (Abcam, ab 6885, Cambridge, UK) was added at 2 µg/mL for 1 h at room temperature. After washing, the color reaction was developed by adding 50 µL of TMB liquid substrate (T0440, Sigma-Aldrich, St. Louis). After 30 min, the reaction was stopped by adding 50 µL of Stop Reagent (S5814, Sigma-Aldrich, St. Louis) and the plate was read at 450 nm in a plate reader (FLUOstar Omega, BMG Labtech, Ortenberg, Germany).

### Collectin-Based Binding Assay

hSP-A was immobilized in a black 96-well plate (655076, Greiner BIO-One, Frickenhausen, Germany) by coating the plate with a specific anti-SP-A antibody (Abcam, abab190087, Cambridge, UK) at a concentration of 0.5 µg/mL in coating buffer [0.1 M NaHCO_3_ buffer (pH 9.6)] at 4°C overnight. Afterward, the plate was blocked with 100 µL Synblock (Bio-Rad, BUF034B, California) for 2 h at room temperature. Then, hSP-A was added at 14 ng/well and incubated with the antibody for 1 h at room temperature in washing buffer (5 mM Tris, 0.9% NaCl, and 0.05% Tween-20) to immobilize it. After washing with washing buffer, different concentrations of HA or HS ranging from 10 mM to 1 nM were added in either washing buffer without calcium or with 5 mM CaCl_2_ and incubated with the immobilized protein for 1 h at room temperature. The following washing steps were performed with washing buffer containing no calcium or 5 mM CaCl_2_. To detect the GAGs bound to the protein, an AlexaFluor488-conjugated wheat germ agglutinin (WGA, W11261, Invitrogen, Massachusetts) was added at 50 µg/mL in washing buffer containing no calcium or 5 mM CaCl_2_ and incubated it for 30 min at room temperature protected from light. After washing with washing buffer with or without calcium, 100 µL of washing buffer without calcium was added to read the fluorescence of the bound WGA with an excitation filter at 485 nm and an emission filter at 520 nm in a plate reader (FLUOstar Omega, BMG Labtech, Ortenberg, Germany).

### DMMB Assay for GAG Quantification

The ability of the dimethylmethylene blue (DMMB) assay to detect GAGs is based on the phenomenon of metachromasia, with the characteristic blue of the cationic DMMB dye shifting to violet when the dye binds to polyanionic substrates such as GAGs (43). We adapted this protocol from previously published ones (44, 45). The DMMB reagent was prepared at pH 3 with 16 mg/L of DMMB (Serva Electrophoresis GmbH, 20335.01, Heidelberg, Germany), glycine (3.05 g/L), NaCl (1.6 g/L), and 544 µL of glacial acetic acid. The standard curve was prepared with different concentrations of chondroitin sulfate (456280050, Acros Organics, Beel, Belgium) in 0.9% NaCl ranging from 100 µg/mL to 0 µg/mL. Twenty microliters of the standards or undiluted BAL fluid samples were used per well in triplicate and 150 µL of the DMMB reagent was subsequently added. The absorbance at 525 nm was immediately measured in a plate reader (FLUOstar Omega, BMG Labtech, Ortenberg, Germany). To correct for potential differences in the dilution of the collected BAL fluid from the animals, the amount of total protein in undiluted BAL fluid was quantified using the BCA assay kit (23225, ThermoFisher, Henningsdorf, Germany) and this value was then used to normalize the GAG amount by total protein amount.

### Alcian Blue Staining and Transmission Electron Microscopy of Lung Tissue

For histological experiments, mouse lungs were fixed with 1.5% paraformaldehyde (PFA) and 1.5% glutaraldehyde (GA) in HEPES (150 mM, pH 7.3) buffer after euthanizing the animal and perfusion of the vascular system (with 0.9% NaCl). Lungs were then isolated and further fixed through immersion in the same fixing solution for a minimum of 24 h at 4°C. Following fixation, lung tissue was cut into small pieces, which were afterward gently massaged with a wooden skewer in Alcian blue 8GX (A5268, Sigma-Aldrich, St. Louis) solution (0.15%, pH 3) and further incubated in the same solution overnight. Next, pieces of lung tissue were osmicated with 1% OsO_4_ (Electron Microscopy Sciences, Hatfield) in 0.1 M cacodylate buffer for 2 h at room temperature (RT) followed by incubation in half-saturated (4%) aqueous uranyl acetate (Merck, Burlington) overnight at 4°C. Afterward, samples were embedded in EPON. Ultrathin sections of 70 nm were cut using an ultramicrotome (Leica, Wetzlar, Germany) equipped with a diamond knife (Diatome, Nidau, Switzerland), collected on pioloform-coated copper grids, and subsequently stained with lead citrate as previously described (46). Visualization was performed with a Zeiss Leo 906 electron microscope at 80 kV acceleration voltage, equipped with a slow scan 2 K CCD camera (TRS, Moorenweis, Germany).

### Data Analysis

Every set of 16 capillaries was prepared in 3–6 replicates (technical replicates) and measured. The replicate measures of every capillary were plotted against concentration and outliers were excluded, using the MO Affinity Analysis 3 v01 software (NanoTemper Technologies, Munich, Germany). K_D_ confidence (indicating 68% certainty for the range in which the K_D_ falls) and response amplitude were calculated by the MO Affinity Analysis 3 v01 software. The raw data were exported to Microsoft Excel and the binding affinity fitting was performed with GraphPad Prism 9.5.1 software. The binding affinity curve was therefore fitted to 3–6 points per concentration. One curve fitting was performed per sugar or GAG. The raw data were fitted (expressed as fraction bounded) to the “non-linear regression one site-specific binding model” (assuming binding saturation and including a constrain B_max_ = 1) and calculated an apparent K_D_ for each carbohydrate at a protein concentration of 9 nM.

The data of the binding inhibition assay were analyzed in GraphPad Prism 9.5.1 using the “normalize” function and fitting to the nonlinear regression dose-response inhibition. The best fitting was achieved using the “[Inhibitor] versus response–variable slope (four parameters)” model. The fluorescence values from the collectin-based binding assay were normalized using the same “normalize” function in GraphPad, and the statistical test used was the “non-parametric Kruskal-Wallis” test. The statistical test used for the DMMB assay for GAG quantification was also the “non-parametric Kruskal-Wallis” test.

## RESULTS

**Figure 1.**
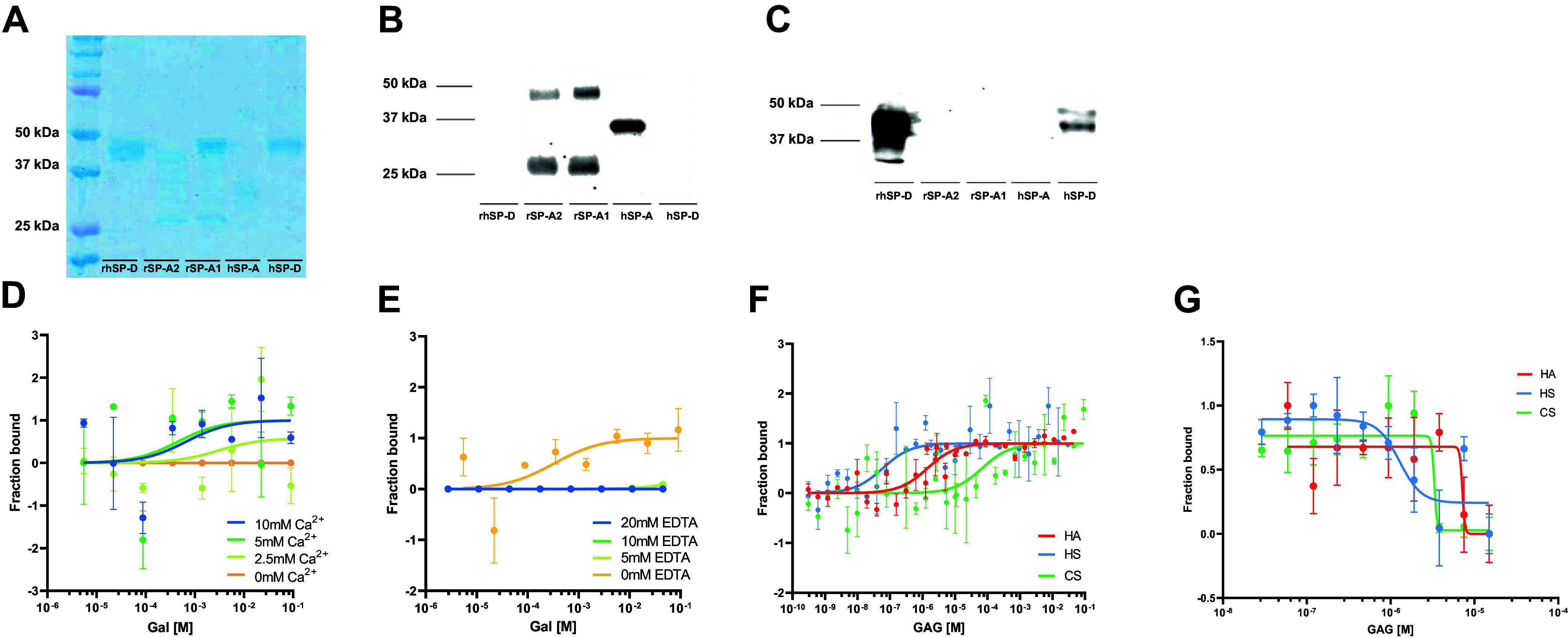
*A*: SDS-PAGE and Coomassie blue staining. *B*: Western blot with anti-SP-A antibody. *C*: Western blot with anti-SP-D antibody of the protein samples used in this study. *D*: binding curves of hSP-A to galactose in the absence of calcium (0 mM calcium, orange) or presence of 2.5 mM (light green), 5 mM (green), and 10 mM calcium (blue). *E*: binding curves of hSP-A to galactose in the presence of 5 mM calcium and absence of EDTA (0 mM EDTA, orange) or presence of 5 mM (light green), 10 mM (green), or 20 mM EDTA (blue). *F*: binding curve of hSP-A to HA (red), HS (blue), and CS (green) at the MST. *G*: binding inhibition curve of hSP-A to mannan in the presence of HA (red), HS (blue), and CS (green). Data are presented as means ± SEM of an *n* = 3–6 technical replicates. CS, chondroitin sulfate; HA, hyaluronan; HS, heparan sulfate; hSP-A, human SP-A; MST, microscale thermophoresis; SP-A, surfactant protein A.

The isolated and recombinant proteins used here were subjected to gel electrophoresis and Western blot, for assessment of purity and specificity. Figure 1*A* shows the proteins present in our samples and the immune detection of SP-A (Fig. 1*B*) and SP-D (Fig. 1*C*) in Western blot. Isolated human proteins showed a band at the expected size of the protein and specificity was positive for SP-A (Fig. 1*B*) using an anti-hSP-A antibody, and SP-D (Fig. 1*C*) using an anti-hSP-D antibody. Traditionally, inhibition assays or affinity columns are used to measure the binding affinity of SP-A and SP-D to carbohydrates (18, 19, 41, 42, 47, 48). Here, we first revisited this binding with microscale thermophoresis (MST), a method that allowed us to prepare and analyze 16 different concentrations of ligands with a minimal amount of protein. To validate the MST method, we first tested the known binding affinity of human SP-A (hSP-A) to galactose in different conditions. As CTL, the binding of SP-A to sugars has been described to be calcium-dependent and starting at a calcium concentration of 1 mM (49). As seen in Fig. 1*D*, the binding of hSP-A to galactose was only seen in the presence of calcium and absence of calcium-chelating agents, such as EDTA (Fig. 1*E*). Following this validation, we systematically analyzed the binding of hSP-A and rhSP-D to different monosaccharides, disaccharides, and amino sugars. The different binding K_D_ for the different sugars are presented in Tables 3 and 4 and show values in the mM range for all of them, including amino sugars. Compared with the published data, our calculated binding affinity seems to be one order of magnitude higher, however, inhibition concentrations and dissociation constants are not directly comparable. Moreover, while IC_50_ values are assay dependent, K_D_ values represent universal constants and thus are assay-independent.

**Table 3.** Data available in the literature for human SP-A binding affinity to sugars compared with MST measurements

Sugar	Amount of SP-A bound (Haagsman et al.; 18)	IC_50_ (Haurum et al.; 41)mean (mM) ±SD	K_D_ (MST Measurements) ±Kd confidence (mM)
Man	95 ± 10%	39 ± 3	0.14 (±0.23)
Glc	100%	30 ± 1	1.39 (±2.36)
Fuc	100%	22 ± 1	4.07 (±4.74)
Maltose		23 ± 5	0.03 (±0.04)
Gal	100%		1.94 (±4.59)
GlcAc			0.09 (±0.07)
ManNAc		8 ± 1	0.06 (±0.25)
GalNAc	7%		7.27 (±12.45)
GlcNAc	2%		0.16 (±0.36)
Neu5Ac			0.21 (±0.42)

IC_50_ refers to the concentration at which half of the protein is bounded to the ligand under the presence of an inhibitor. K_D_ is the equilibrium dissociation constant, indicating the concentration at which half of the protein is bound directly to the ligand.

**Table 4.** Data available in the literature for human SP-D binding affinity to sugars compared with MST measurements

Sugar	I_50_ (Persson et al.; 48) Absorbance Relative to Control	IC_50_ (Lu et al.; 19)mean (mM)	IC_50_ (van Eijk et al.; 42)* mean (mM) ± SE	I_50_ (Crouch et al. 2006)** mean (mM) ± SE	K_D_ (MST Measurements) ±Kd confidence (mM)
Man	25	7.5	2.9 ± 0.4	5.3 ± 0.5	0.02 (±0.08)
Glc	8.5	9.6	4.0 ± 1.0	4.1 ± 0.9	0.12 (±0.17)
Fuc	31	6.5	4.4 ± 0.1		0.37 (±0.71)
Maltose	3.4	5.1	4.1 ± 0.9	2.7 ± 0.4	0.45 (±0.56)
Gal	29	22	7.9 ± 3.8	10 ± 2	0.82 (±1.63)
GlcAc	22				0.005 (±0.007)
ManNAc			1.8 ± 0.3	2.1 ± 0.4	5.39 (±13.86)
GlcNAc	87	28.5	13.5 ± 4.5	14 ± 2	0.13 (±0.31)
GalNAc	>100				0.39 (±0.69)
Neu5Ac					0.27 (±0.31)

I_50_ or IC_50_ refers to the concentration at which half of the protein is bounded to the ligand under the presence of an inhibitor. K_D_ is the equilibrium dissociation constant, indicating the concentration at which half of the protein is bound directly to the ligand. *Data in van Eijk et al. (42) comes from a recombinant human SP-D. **Data in Crouch et al. (47) comes from a recombinant construct containing the N-terminal tagged fusion protein +neck and CRD of human SP-D. CRD, carbohydrate recognition domain; SP-D, surfactant protein D.

As there are no data available in the literature on the binding affinity of SP-A or SP-D to GAGs, such as HA, HS, and CS, we first performed a complete titration curve of the GAGs, from the molar to nanomolar range, to understand the range in which we should analyze the binding in more detail. Figure 1*F* shows the fitting of the binding of hSP-A to the different concentrations of the GAGs, confirming that hSP-A is able to bind to all GAGs tested. Based on these data, we focused on the concentration range of GAGs between 10^−5^ and 10^−10^ M for further analysis. To confirm the binding of hSP-A to GAGs, we also performed an adapted inhibition assay, in which a microtitre plate is coated with mannan and the binding of hSP-A is inhibited by adding different concentrations of GAGs. hSP-A binding is detected with an anti-hSP-A antibody. By use of this biochemical binding competition assay, we probed our three GAG representatives (HA, HS, and CS) for their capability to displace hSP-A from its complex with mannan. Figure 1*G* shows the binding inhibition curves of hSP-A to HA, HS, and CS. All three GAGs demonstrated hSP-A binding inhibition to mannan in the µM range supporting the data obtained by MST.

**Figure 2.**
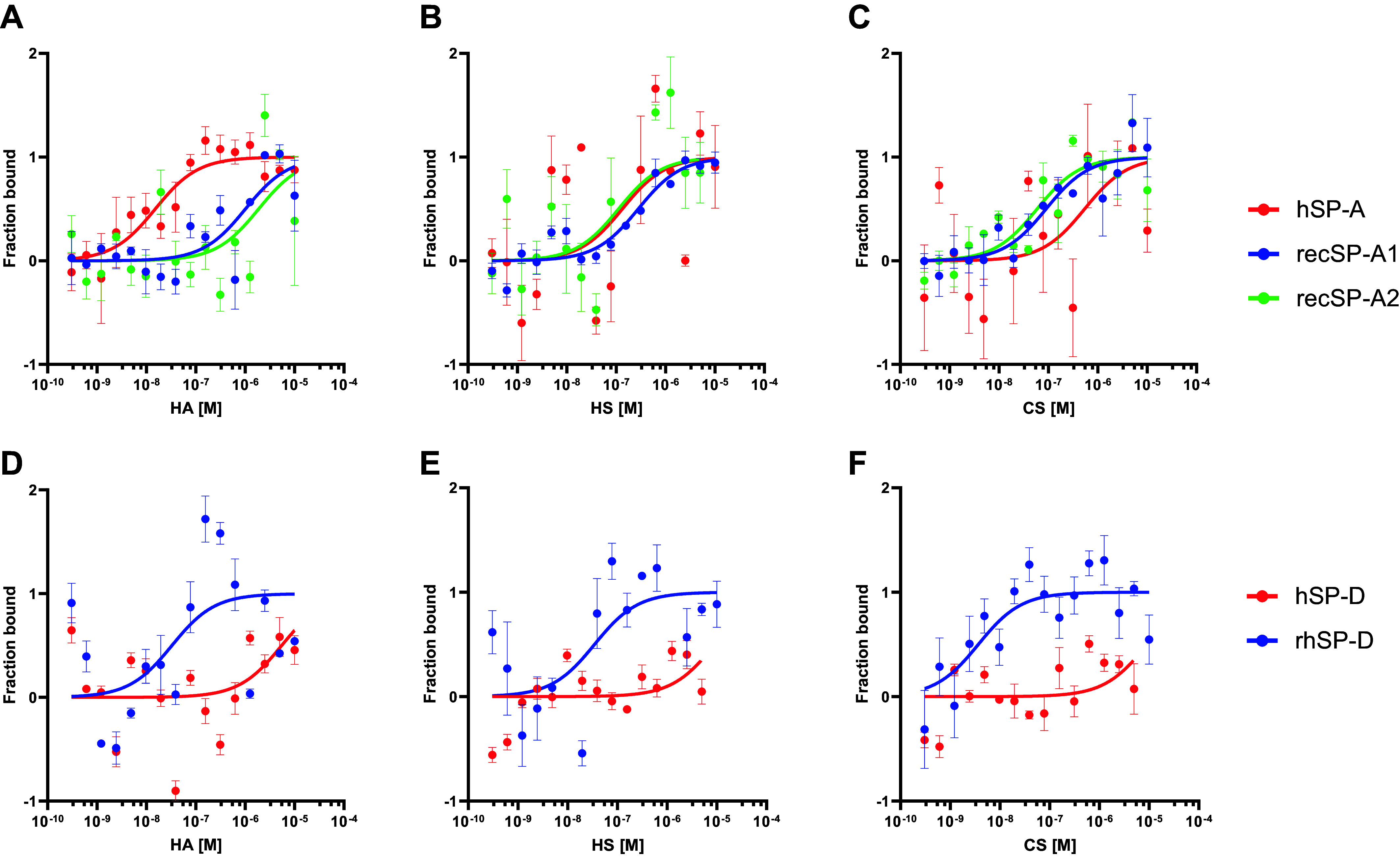
Binding curves measured at the MST of SP-A and SP-D of different origin to GAGs. *Top*: binding of hSP-A (red), recSP-A1 (blue), and recSP-A2 (green) to: HA (*A*), HS (*B*), and CS (*C*). *Bottom*: binding of hSP-D (red) and rhSP-D (blue) to: HA (*D*), HS (*E*), and CS (*F*). Data are presented as means ± SEM of an *n* = 3–6 technical replicates. CS, chondroitin sulfate; GAG, glycosaminoglycan; HA, hyaluronan; HS, heparan sulfate; hSP-A, human SP-A; MST, microscale thermophoresis; SP-A, surfactant protein A; SP-D, surfactant protein D.

After validation of MST to measure the binding of the surfactant proteins to GAGs, we compared the binding affinity to GAGs of hSP-A and two recombinant human proteins, SP-A1 and SP-A2. All proteins showed (Fig. 2, *A*–*C*) an apparent binding in the µM–nM range (Table 5). In the next step, we compared the binding affinity of human SP-D (hSP-D) and rhSP-D to GAGs (Fig. 2, *D* and *E*). In case of SP-D, both proteins show the same pattern, with an apparent binding to GAGs again in the µM–nM range (Table 5). This higher affinity, of 2–4 orders of magnitude in K_D_ values, relative to the binding affinity to monosaccharides-, disaccharides, and amino sugars (Tables 3 and 4) is not just explained by the several binding sites present on a GAG molecule so some cooperativity effect may occur between them and multiple units in oligomeric forms of hSP-A or rhSP-D.

**Table 5.** Calculated K_D_ (M) with K_D_ confidence and response amplitude in the MST measurements for all the proteins used in this study and the GAGs from Fig. 2

hSP-A	K_d_ (±K_D_ Confidence) (M)	Response Amplitude
HA	1.22 × 10^−08^ (±0.79)	12.93
CS	2.25 × 10^−07^ (±4.19)	8.37
HS	2.33 × 10^−08^ (6.09)	11.92

recSP-A1	K_d_ (±K_D_ Confidence) (M)	Response Amplitude
HA	1.01 × 10^−06^ (±0.97)	17.13
CS	8.01 × 10^−08^ (±4.49)	17.02
HS	2.69 × 10^−07^ (±1.28)	15.51

recSP-A2	K_d_ (±K_D_ Confidence) (M)	Response Amplitude
HA	1.87 × 10^−06^ (±3.54)	19.28
CS	5.52 × 10^−08^ (±4.12)	31.90
HS	1.43 × 10^−07^ (±3.09)	15.59

hSP-D	K_d_ (±K_D_ Confidence) (M)	Response Amplitude
HA	2.19 × 10^−06^ (±1.82)	3.45
CS	5.82 × 10^−06^ (±3.91)	3.74
HS	5.64 × 10^−06^ (±4.21)	3.03

rhSP-D	K_d_ (±K_D_ Confidence) (M)	Response Amplitude
HA	2.21 × 10^−08^ (±5.33)	9.67
CS	8.08 × 10^−10^ (±0.19)	23.54
HS	2.87 × 10^−07^ (±0.43)	3.55

CS, chondroitin sulfate; HA, hyaluronan; HS, heparan sulfate.

To test this new notion, we next investigated the presence of higher oligomeric forms of the proteins used in this study by interferometric scattering microscopy (iSCAM) (Table 6) and found high oligomeric forms in all proteins with higher proportions in the human-isolated proteins and rhSP-D compared with recSP-A1 and recSP-A2.

**Table 6.** Detection of the molecular assembly states of different surfactant proteins from iSCAM measurements

	MW from GE	MW Form iSCAM			
Protein	Monomer	Species 1	Species 2	Species 3	Species 4
recSP-A1	54 kDa	43% (63 kDa)	14% (102 kDa)	8% (141 kDa)	5% (207 kDa)
recSP-A2	39 kDa	22% (42 kDa)	20% (85 kDa)	13% (125 kDa)	8% (197 kDa)
recSP-D	43 kDa	8% (54 kDa)	31% (113 kDa)	4% (234 kDa)	33% (462 kDa)
hSP-A	36 kDa	27% (64 kDa)	10% (147 kDa)	30% (495 kDa)	14% (1,009 kDa)
hSP-D	43 kDa	14% (57 kDa)	23% (113 kDa)	12% (254 kDa)	28% (456 kDa)

Percentage values represent counts within the Gaussian fit of each molecular weight population in proportion to the total counts of three measurements. GE, gel electrophoresis; iSCAM, interferometric light scattering microscopy; MW, molecular weight.

**Figure 3.**
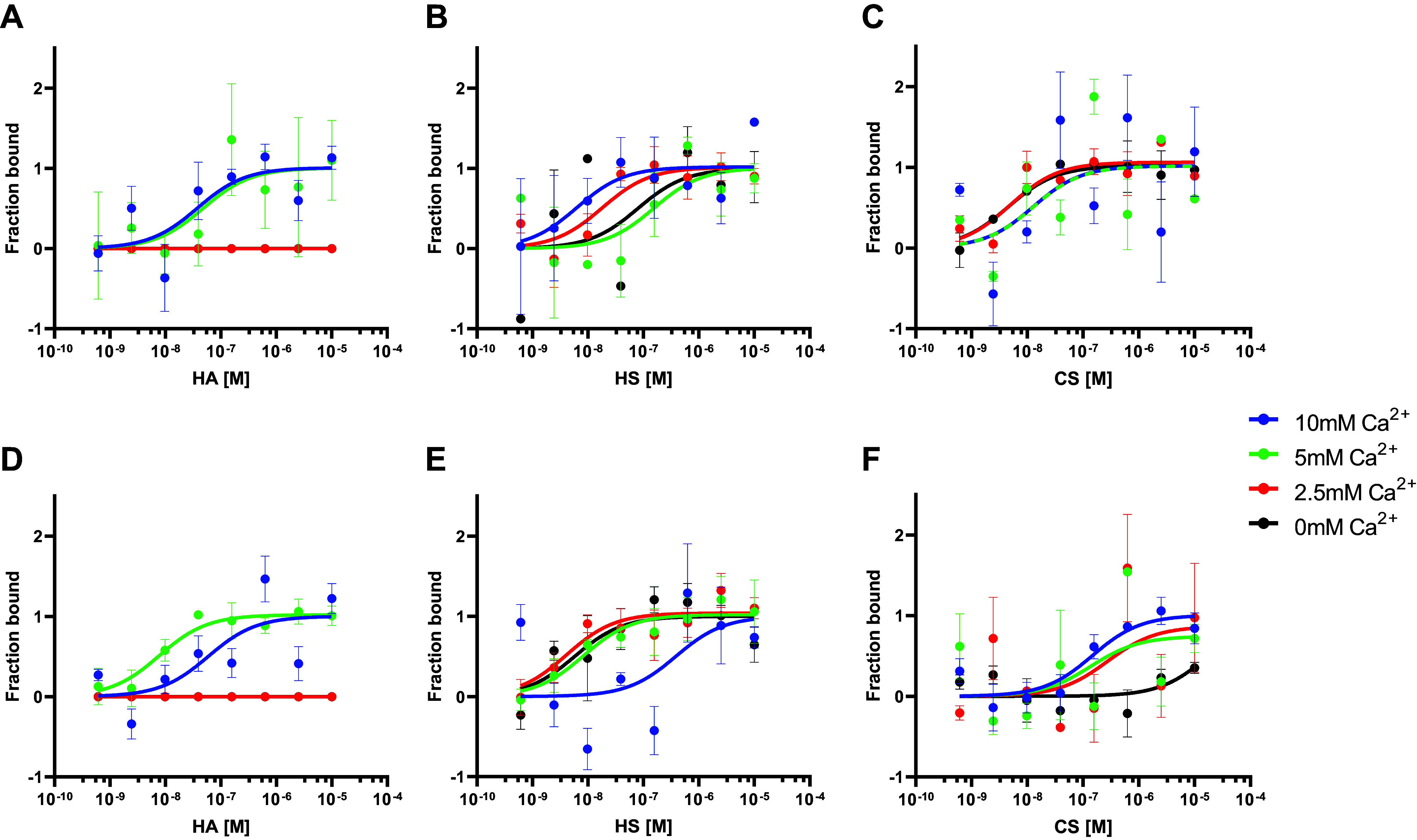
Binding curves of hSP-A and rhSP-D to GAGs at different calcium concentrations. *Top*: binding curves of hSP-A in the presence of 0 mM (black), 2.5 mM (red), 5 mM (green), and 10 mM (blue) calcium to HA (*A*), HS (*B*), and CS (*C*). *Bottom*: binding curves of rhSP-D in the presence of 0 mM (black), 2.5 mM (red), 5 mM (green), and 10 mM (blue) calcium to HA (*D*), HS (*E*), and CS (*F*). Data are presented as means ± SEM of an *n* = 3–6 technical replicates. CS, chondroitin sulfate; GAG, glycosaminoglycan; HA, hyaluronan; HS, heparan sulfate; hSP-A, human SP-A.

To our knowledge, this is the first report showing the binding of both SP-A and SP-D to model glycosaminoglycans. To better characterize the interaction of SP-A and SP-D with GAGs, we next investigated the effect of varying calcium concentrations on the binding properties. As shown in Fig. 3*A*, hSP-A was not able to bind to HA in the absence or presence of low calcium concentrations. Next, we tested if binding to sulfated GAGs is similarly dependent on calcium. Figure 3, *B* and *C,* shows that hSP-A binds to HS and CS also at low, or even in the absence of calcium. This phenomenon was reproduced when we tested rhSP-D for binding to HA (Fig. 3*D*) and HS and CS (Fig. 3, *E* and *F*). Corresponding K_D_ affinity values are given in Table 7.

**Table 7.** Calculated K_D_ (M) with K_D_ confidence and response amplitude in the MST measurements for all the proteins used in this study and the GAGs at different calcium concentrations from Fig. 3

hSP-A + HA	K_D_ (M)	K_D_ Confidence	Response Amplitude
0 mM Ca^2+^			
2.5 mM Ca^2+^			
5 mM Ca^2+^	2.91 × 10^−09^	1.88 × 10^−09^	7.31
10 mM Ca^2+^	5.46 × 10^−08^	1.08 × 10^−07^	3.65

hSP-A + HS	K_D_ (M)	K_D_ Confidence	Response Amplitude
0 mM Ca^2+^	3.22 × 10^−09^	6.57 × 10^−09^	3.61
2.5 mM Ca^2+^	1.88 × 10^−10^	6.98 × 10^−10^	9.27
5 mM Ca^2+^	3.74 × 10^−09^	3.85 × 10^−09^	7.51
10 mM Ca^2+^	3.75 × 10^−07^	1.16 × 10^−06^	2.79

hSP-A + CS	K_D_ (M)	K_D_ Confidence	Response Amplitude
0 mM Ca^2+^	9.99 × 10^−10^	8.68 × 10^−10^	7.16
2.5 mM Ca^2+^	1.19 × 10^−11^	2.40 × 10^−10^	13.45
5 mM Ca^2+^	7.38 × 10^−09^	2.71 × 10^−08^	8.07
10 mM Ca^2+^	5.91 × 10^−09^	2.67 × 10^−08^	6.89

rhSP-D + HA	K_D_ (M)	K_D_ Confidence	Response Amplitude
0 mM Ca^2+^			
2.5 mM Ca^2+^			
5 mM Ca^2+^	4.19 × 10^−08^	6.51 × 10^−08^	5.33
10 mM Ca^2+^	3.11 × 10^−08^	5.58 × 10^−08^	3.25

rhSP-D + HS	K_D_ (M)	K_D_ Confidence	Response Amplitude
0 mM Ca^2+^	7.35 × 10^−07^	1.34 × 10^−06^	12.04
2.5 mM Ca^2+^	7.44 × 10^−09^	6.06 × 10^−09^	33.47
5 mM Ca^2+^	4.31 × 10^−07^	8.03 × 10^−07^	6.29
10 mM Ca^2+^	6.88 × 10^−10^	9.81 × 10^−10^	15.22

rhSP-D + CS	K_D_ (M)	K_D_ Confidence	Response Amplitude
0 mM Ca^2+^	1.76 × 10^−05^	1.33 × 10^−04^	16.10
2.5 mM Ca^2+^	1.52 × 10^−07^	7.18 × 10^−07^	10.05
5 mM Ca^2+^	9.71 × 10^−08^	4.20 × 10^−07^	10.83
10 mM Ca^2+^	1.31 × 10^−07^	1.16 × 10^−07^	21.36

CS, chondroitin sulfate; HA, hyaluronan; HS, heparan sulfate.

**Figure 4.**
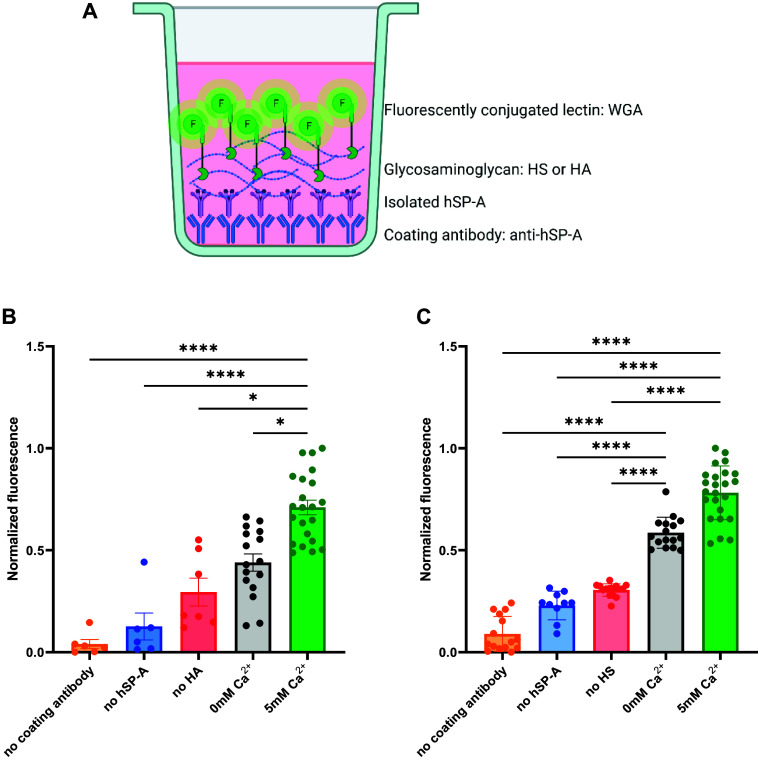
Collectin-based binding assay of hSP-A to HA and HS in the presence or absence of calcium. *A*: scheme of the method with immobilized hSP-A binding to GAGs, which are then detected by a fluorescently conjugated lectin. *B*: normalized fluorescence of WGA bound to HA in the absence (black) or presence (green) of calcium, compared with technical controls in the absence of coating antibody (orange), or hSP-A (blue) or HA (red). *C*: normalized fluorescence of WGA bound to HS in the absence (black) or presence (green) of calcium, compared with technical controls in the absence of coating antibody (orange), or hSP-A (blue) or HA (red). Data are presented as means ± SD of an *n* = 8–16 technical replicates, statistical significance was assessed with a non-parametric Kruskal–Wallis test considering *P < 0.05 and ****P < 0.0001 values. GAG, glycosaminoglycan; HA, hyaluronan; HS, heparan sulfate; hSP-A, human SP-A. [Image *A* created with a licensed version of BioRender.com.]

We next aimed to validate this calcium-independent binding by a second approach. The previously used binding inhibition assay (Fig. 1*G*) is based on the calcium-dependent binding of surfactant collectins to mannan, and hence, does not allow us to assess the effects of varying calcium concentrations. Assuming that surfactant collectins bind to GAGs, we developed a collectin-based fluorescent method to confirm their binding ability. As shown in Fig. 4*A*, we immobilized hSP-A by specific antibodies similar to ELISA-based methods. After incubation with the isolated human protein, which binds specifically to the antibody, we exposed the immobilized protein to either HA or HS in the presence or absence of calcium and their corresponding technical negative controls. To detect the attached GAG, we used a fluorescently conjugated wheat germ agglutinin (WGA) that binds to GlcNAc, which is present in both HA and HS. Normalized fluorescence data are shown in Fig. 4, *B* and *C*. These results showed increased fluorescence in the absence of calcium when hSP-A was exposed to HS, yet no significant increase in fluorescence when HA binding was tested. In the presence of 5 mM calcium, however, hSP-A was again able to bind to both HA and HS in this assay as shown by the respective increase in normalized fluorescence. The technical negative controls performed here account for the potential binding of WGA to sialic acid residues in either the antibody, the protein, or both. These results support the finding from our MST measurements that SP-A binding to sulfated GAG is calcium-independent.

**Figure 5.**
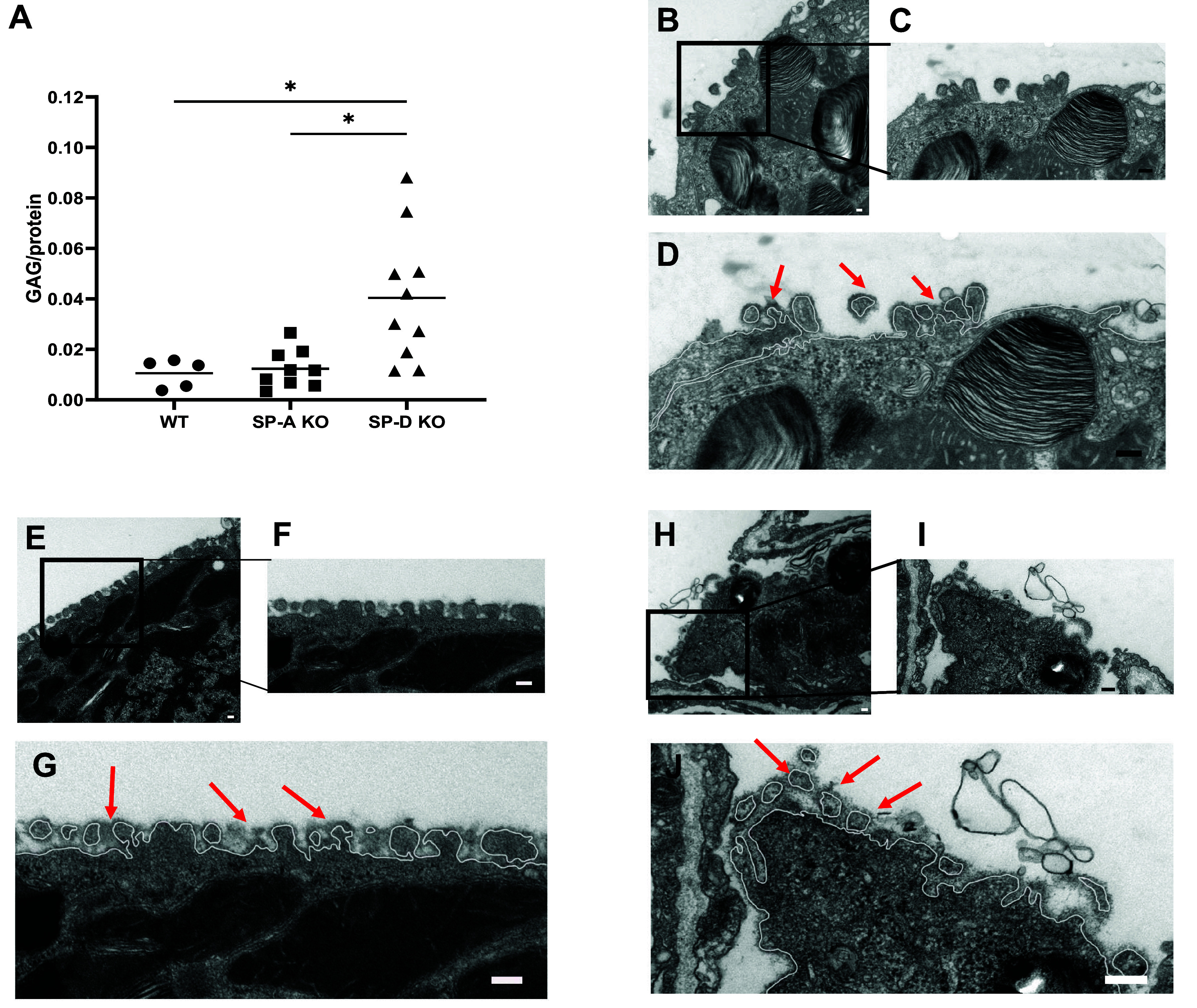
*A*: quantification of GAGs in BAL from WT, SP-A KO, and SP-D KO mice, corrected by total protein in BAL. Alcian blue stained surface of microvilli of AE2C (*B*), higher magnification picture (*C*), and white line representing cell membrane and stained glycocalyx on top (red arrows) in WT mice (*D*). Alcian blue stained surface of microvilli of AE2C (*E*), higher magnification picture (*F*), and white line representing cell membrane and stained glycocalyx on top (red arrows) in SP-A KO mice (*G*). Alcian blue stained surface of microvilli of AE2C (*H*), higher magnification picture (*I*), and white line representing cell membrane and stained glycocalyx on top (red arrows) in SP-D KO mice (*J*). Data are presented as means ± SD of an *n* = 8–16 technical replicates, statistical significance was assessed with a non-parametric Kruskal–Wallis test considering *P < 0.05. Scale bar = 100 nm. BAL, bronchoalveolar lavage; GAG, glycosaminoglycan; SP-A, surfactant protein A; SP-D, surfactant protein D.

To understand the potential physiological relevance of these interactions at the alveolar glycocalyx, we quantified the amount of GAGs in BAL fluid of mice lacking either SP-A (SP-A KO) or SP-D (SP-D KO). In the recovered BAL fluid, we can collect free or shedded components of the alveolar glycocalyx, such as HS, HA, and CS. As shown in Fig. 5*A*, in the absence of SP-D, we found the amount of GAGs in the BAL fluid to be significantly increased.

However, the amount of total GAG is not exclusively related to the interaction of lung collectins with the extracellular glycocalyx components but is also regulated by other mechanisms. Thus, we directly probed for differences between SP-A and SP-D deficient and corresponding wild-type (WT) mice lungs at the ultrastructural level of the alveolar epithelial glycocalyx in vivo.

Figure 5, *B*–*D*, shows representative electron micrographs of the surface of an alveolar epithelial type II cell with typical microvilli. Figure 5*D* shows an electron densely stained layer (red arrows) around microvilli, on top of the cell membrane (white line), in alveolar epithelial type 2 cells (AE2C) of WT mice, corresponding to Alcian blue binding to negative charges from glycan components of the alveolar epithelial glycocalyx. Figure 5, *E*–*G,* shows a similar staining pattern in the SP-A deficient mice. In contrast, Fig. 5, *H*–*J,* shows a more dispersed and less intense electron-dense layer (red arrows) at the surface of microvilli in AE2C of SP-D KO mice, indicating that collectins may play an important role in regulating the alveolar epithelial glycocalyx structure at the alveolar epithelial surface.

## DISCUSSION

Here, we assessed the binding of SP-A and SP-D to monosaccharides, disaccharides, and GAGs using MST for the first time. With this method, titration curves of 16 different ligand concentrations can be obtained within one experiment with a minimal amount of protein (9 nM). In addition, binding affinity dissociation constants (K_D_) are obtained that directly relate to the formation of the protein-ligand complex. So far, binding of surfactant collectins has been traditionally assessed by inhibitory (competitive) assays and exclusively for monosaccharides and disaccharides. These assays are originally derived from the method used to measure the binding affinity of mannose-binding protein (MBP) to mannan, a linear polysaccharide of repetitive units of mannose (50, 51), which may resemble the binding to other GAGs. Differences and discrepancies between assays measuring the binding affinities or binding inhibition have been previously discussed in detail (41, 52). Besides differences in sensitivity of the different methods used in the literature to analyze the binding of SP-A and SP-D to monosaccharides and disaccharides, results may in addition vary based on the source of the proteins, i.e., human or recombinant proteins, studied previously and in this report.

Even when we were able to calculate for the first time K_D_ for the binding of SP-A and SP-D with MST, the method has also several distinct limitations. To perform the experiments, we labeled the proteins with fluorescence tags, which may introduce conformation modifications or differences in binding ability. Specifically, we labeled the lysine residues of the proteins, which means that four lysine residues were labeled within the CRD domain in SP-A (out of 8 total lysines in the sequence of the protein) and eight lysines in the CRD domain of SP-D (out of 21 lysines in the sequence of the protein). This may have an effect on the carbohydrate recognition ability of the proteins, resulting in over- or underestimation of our K_D_ calculations. A further source of potential discrepancies is the source of the protein. Of note, in most studies, human SP-A and SP-D are isolated from BAL fluid of patients with pulmonary alveolar proteinosis (PAP), as these BAL fluid samples yield a high amount of these surfactant collectins due to the surfactant accumulation in the lungs of these patients. Whether these SP-A and SP-D can be considered “normal or healthy controls” is questionable. Clinical PAP is mainly caused by impairment of macrophage-dependent surfactant removal, e.g., due to the presence of antibodies against the granulocyte-macrophage colony-stimulating factor (GM-CSF) or mutations in the genes for GM-CSF. No differences in the elevated concentrations of SP-A have been identified between the different forms of PAP (37). However, little is known about the posttranslational processing of surfactant proteins in PAP, and whether this is comparable to healthy individuals or not. For example, surfactant collectins of patients with PAP may present different glycosylation patterns as compared with healthy controls (53), which may in turn interfere with their sugar binding. Along these lines, it has already been described that different glycosylation patterns of recombinant SP-D in the CRD impair the protein binding to different pathogens (54, 55). On the other hand, recombinant proteins may also vary from human samples and between each other as a function of source (Table 1), sequence, oligomeric (Table 6), and glycosylation status. Variations in glycosylation could explain the differences in binding affinity to GAGs observed here for the human and human recombinant SP-D (Table 5 and Fig. 2) as oligomerization seems to be similar (Table 6). On the other hand, SP-A1 and SP-A2 variants do not show any significant difference in GAG binding, as opposed to what could be hypothesized from previous suggestions (49).

To date, there are limited data available addressing the binding of both SP-A and SP-D to GAGs. SP-D has, however, previously been reported to bind to a proteoglycan, namely, decorin. Nadesalingam and colleagues (56) concluded that SP-D binds through the CRD domain to the GalNAc moiety of the glycosaminoglycan chain of human decorin, dermatan sulfate. In addition, Murugaiah and colleagues (57) recently reported the binding of a recombinant fragment of SP-D to HA. Here, we expanded these analyses systematically for other GAGs. We found that both proteins bind with high affinity to the three GAGs investigated here. Compared with the binding affinities to single monosaccharides, typically found in these GAGs, specifically GalNAc, GlcNAc, and GlcAc, which are in the range of mM, the affinity to GAGs falls in the range of µM to nM. This finding is in line with a multivalent binding of an oligomeric lectin, and with previous work demonstrating that single site-binding affinities in many lectins appear to be low (K_D_ within µM range) while multivalent glycans are recognized with higher affinity (K_D_ within nM range) (58). Lectins usually recognize specific terminal moieties of glycan chains by fitting them into shallow, but relatively well-defined, binding pockets. Selectivity is mostly achieved by a combination of hydrogen bonds (involving the hydroxyl group of the sugar) and by van der Waals packing of the hydrophobic face of the monosaccharide ring against aromatic amino acid side chains. The actual region of contact between the saccharide and the protein typically involves 1–3 monosaccharide residues (59). In contrast, protein interaction with, for example, sulfated GAGs seems to involve surface clusters of positively charged amino acids that line up against internal regions of the extended anionic GAG chains (59). Examples of such lectins, which are typically oligomeric and multivalent glycan-binding proteins, include galectins and C-type lectins such as serum collectins (60).

Even though lectins are traditionally not described to bind to GAGs, Toda and colleagues (61) described already in 1981 the binding of lectins such as wheat germ agglutinin (WGA) and *Solanum tuberosum* agglutinin (STA) to sulfated GAGs. In 2016, Talaga and colleagues (62) showed binding of Galectin-3 to sulfated GAGs and CS-proteoglycans, proposing new roles for this protein. Recently, Sandoval and colleagues (63) isolated HS from endothelial cells and analyzed bound proteins using a proteomic-based method. They report for the first time a C-type Lectin (CTL), namely, CLEC14A, as well other proteins (including other CTLs) to bind to sulfated GAGs, such as HS. L- and P-selectins have been shown to bind to a subfraction of HS chains and heparin in a divalent cation-dependent manner. Collectively, these findings suggest that other CTLs may similarly interact with GAG chains, supporting our findings on the binding of surfactant collectins to HA, HS, and CS. The nature of these molecular interactions are out of the scope of this report, but it is thought that GAGs bind to many different classes of proteins mostly through electrostatic interactions between negatively charged sulfate groups and uronic acids and positively charged amino acids in the protein (64).

Very recently, Rizzo and colleagues (9) found direct interaction between HS and surfactant proteins SP-A, SP-B, and SP-D. In addition, they concluded that shedding of HS after lung injury induces surfactant dysfunction, which contributes to the development or progression of the disease. In accordance with their results, we found that both SP-A and SP-D are able to bind HS and other GAGs in a µM to nM range. Both SP-A and SP-D are known to bind to surfactant lipids (65–69), and as such they may interconnect components of lung surfactant to the alveolar epithelial glycocalyx. In addition, SP-A and SP-D have also been shown to bind to other highly negatively charged molecules such nucleic acids (70, 71). In this case, this phenomenon has been related to the binding to DNA to initiate the signaling for removal of dying cells, whether nucleic acids are components of the alveolar epithelial glycocalyx is yet a question to answer (72).

As this interaction of surfactant collectins to GAGs is not well characterized yet, we tested it in different buffer conditions. To elucidate if the binding to SP-A is Ca^2+^-dependent, we performed a series of experiments at varying Ca^2+^ concentrations. Our results (Fig. 3, *A*–*F,* and Fig. 4) indicate that SP-A and SP-D bind to sulfated GAGs in a Ca^2+^-independent way. To date, there is little evidence that CTLs may bind to glycan-containing molecules in the absence of calcium. However, Chabrol and colleagues (73) described already in 2012, the interaction of a CTL from Langerhans cells, Langerin, with GAGs in a calcium-independent manner. These results suggest a different mechanism for sulfated GAGs recognition by surfactant collectins compared with other sugar moieties.

To confirm the potential functional relevance of this newly detected interaction between collectins and GAGs, we probed for the effects of a genetic loss of SP-A or SP-D on the ultrastructural organization of the alveolar epithelial glycocalyx in vivo using established electron microscopy-based visualization methods in our group. With the help of cytochemical staining, we were able to detect differences in the apparent ultrastructure of the glycocalyx in the absence of SP-D. In parallel, the BAL fluid of SP-D KO mice showed higher amounts of total GAGs relative to WT mice (Fig. 5*A*). Taken together, these findings demonstrate differences in the structure and/or organization of the glycan components in the alveolar epithelial glycocalyx in the absence of SP-D, and accordingly, indicate a relevant role of collectins in the composition of the alveolar epithelial glycocalyx under basal, unchallenged conditions. Thus, the increased amounts of GAGs in the SP-D KO mice have an ultrastructural correlate in the Alcian blue staining pattern at the surface of AE2C (Fig. 5*C*). However, cytochemical staining like Alcian blue does not allow a further molecular interpretation. These differences in glycocalyx ultrastructure may have relevant implications for the function of the alveolar epithelial glycocalyx, which should be addressed in future investigations. Interestingly, as mentioned before, SP-D is the least abundant surfactant protein, whose absence has a great impact on the phenotype of the animals, especially related to surfactant recycling and metabolism (74–79). The findings presented here may contribute to understanding the multiple roles of SP-A and SP-D in alveolar homeostasis, integrating the alveolar glycocalyx in the picture.

**Figure 6.**
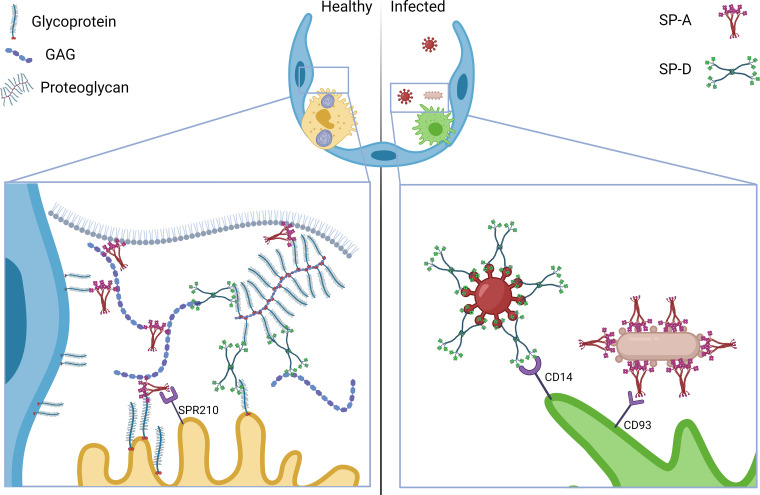
Model of the proposed function of SP-A and SP-D as cross-linking proteins of the alveolar glycocalyx in healthy lung conditions (*right*) where SP-A and SP-D bind GAGs and interconnect free GAGs with proteoglycans and membrane glycoproteins. In addition, SP-A binds to its specific receptor SPR210 on the surface of AE2C and lipids at the air-liquid interface. In contrast, SP-A and SP-D play an important function opsonizing pathogens (*left*). SP-A and SP-D also bind to specific receptors at the surface of alveolar macrophages, such as CD14 and CD93. GAG, glycosaminoglycan; SP-A, surfactant protein A; SP-D, surfactant protein D. [Image created with a licensed version of BioRender.com.]

Taken together, our findings provide evidence for a new important physiological function of surfactant collectins as interaction partners of GAGs, and hence, as potential integral elements of the alveolar epithelial glycocalyx. To date, a large body of data has established the role of collectins for the innate immune response of the lung to pathogens (Fig. 6, *right*), yet their function in the healthy alveolus in the absence of pathogens was so far supposed to be limited to interaction with surfactant lipids (65–69). Here, we propose an alternative model of the alveolar epithelial glycocalyx where, in healthy state, SP-A and SP-D might be interacting with GAGs and other sugar or glycan residues (from membrane integral glycoproteins or glycolipids, proteoglycans, and free proteoglycans) thus interconnecting glycocalyx elements. Due to their parallel ability to bind lipids and cell membrane-specific receptors (80), surfactant collectins may further interconnect surfactant lipids and glycocalyx components to form a stable hydrogel at the alveolar aqueous hypophase or lining layer (Fig. 6, *left*).

## DATA AVAILABILITY

Data will be made available by the authors, upon reasonable request.

## GRANTS

This work has been funded by the Deutsche Forschungsgemeinschaft (DFG, German Research Foundation) within the SFB 1449 – 431232613; sub-projects B01 (to M.O. and W.M.K.) and C04 (to D.L.).

## DISCLOSURES

Wolfgang Kuebler and Matthias Ochs are editors of *American Journal of Physiology-Lung Cellular and Molecular Physiology* and were not involved and did not have access to information regarding the peer-review process or final disposition of this article. An alternate editor oversaw the peer-review and decision-making process for this article. R. A. is currently a paid employee of Airway Therapeutics Inc, which is developing SP-D as human therapeutic agent. None of the other authors has any conflicts of interest, financial or otherwise, to disclose.

## AUTHOR CONTRIBUTIONS

D.L. and E.L.-R. conceived and designed research; R.A., A.V., V.G., N.B., R.A., M.I.-C., and L.G.-O. performed experiments; R.A., A.V., V.G., N.B., D.L., and E.L.-R. analyzed data; D.L. and E.L.-R. interpreted results of experiments; D.L. and E.L.-R. prepared figures; R.A., A.V., V.G., N.B., D.L., and E.L.-R. drafted manuscript; R.A., A.V., V.G., N.B., R.A., P.K., M.I.-C., L.G.-O., W.M.K., M.O., D.L., and E.L.-R. edited and revised manuscript; R.A., A.V., V.G., N.B., R.A., P.K., M.I.-C., L.G.-O., W.M.K., M.O., D.L., and E.L.-R. approved final version of manuscript.
